# Emerging Gene-Editing Modalities for Osteoarthritis

**DOI:** 10.3390/ijms21176046

**Published:** 2020-08-22

**Authors:** Alekya S. Tanikella, Makenna J. Hardy, Stephanie M. Frahs, Aidan G. Cormier, Kalin D. Gibbons, Clare K. Fitzpatrick, Julia Thom Oxford

**Affiliations:** 1 Biomolecular Research Center, Boise State University, Boise, ID 83725, USA; atlearning2014@gmail.com (A.S.T.); makennahardy@u.boisestate.edu (M.J.H.); StephanieTuft@boisestate.edu (S.M.F.); 2 Department of Biological Sciences, Boise State University, Boise, ID 83725, USA; 3 Biomolecular Sciences Graduate Programs, Boise State University, Boise, ID 83725, USA; 4 Mechanical and Biomedical Engineering, Boise State University, Boise, ID 83725, USA; aidancormier@u.boisestate.edu (A.G.C.); kalingibbons@u.boisestate.edu (K.D.G.); clarefitzpatrick@boisestate.edu (C.K.F.)

**Keywords:** osteoarthritis, CRISPR/Cas9, miRNA, genome editing

## Abstract

Osteoarthritis (OA) is a pathological degenerative condition of the joints that is widely prevalent worldwide, resulting in significant pain, disability, and impaired quality of life. The diverse etiology and pathogenesis of OA can explain the paucity of viable preventive and disease-modifying strategies to counter it. Advances in genome-editing techniques may improve disease-modifying solutions by addressing inherited predisposing risk factors and the activity of inflammatory modulators. Recent progress on technologies such as CRISPR/Cas9 and cell-based genome-editing therapies targeting the genetic and epigenetic alternations in OA offer promising avenues for early diagnosis and the development of personalized therapies. The purpose of this literature review was to concisely summarize the genome-editing options against chronic degenerative joint conditions such as OA with a focus on the more recently emerging modalities, especially CRISPR/Cas9. Future advancements in novel genome-editing therapies may improve the efficacy of such targeted treatments.

## 1. Introduction

Osteoarthritis (OA) is a chronic disease affecting the joints of the body, especially the weight-bearing joints, chiefly the knees, hips, shoulders, and spine. OA is the most common type of degenerative arthritis, affecting over 30 million Americans [1]. It is also the second most expensive disease to treat in the US, costing $139.8 billion in 2013 [2]. As the fifth leading cause of disability in the US, OA has a significant impact on the quality of life.

In 2017, the Centers for Disease Control (CDC) published results on the prevalence of doctor-diagnosed arthritis from 2013 to 2015, including 54.4 million adults, nearly half of whom had activity limitations attributable to arthritis [3]. More alarmingly, as our US population ages, the number of cases is projected to increase to 78.4 million, or 25.9% of the population, by 2040 [4]. According to the World Health Organization (WHO), the United Nations has categorized OA as a priority disease in need of research into potential therapies. Given that between 2015 and 2050, the proportion of the world’s population over 60 years old will nearly double from 12% to 22%, an estimated 130 million people will suffer from OA worldwide (WHO, 2018).

The progression of OA includes the degradation of the hyaline articular cartilage at the ends of the articulating bones of the synovial joints. The articular cartilage, when healthy, functions to resist compression, prevent bone–bone contact, and maintain a low-friction surface [5]. Damaged joint articular cartilage, on the other hand, cannot perform the necessary functions [6]. Additionally, the repair of the damaged articular cartilage is a challenging issue, due in part to its avascular nature and its limited ability to heal by itself [5,6,7]. This often results in the formation of fibrocartilage, which lacks the ideal biomechanical characteristics needed to withstand the compressive stress imposed on the synovial joints during articulation and load-bearing [8,9].

**Figure 1:**
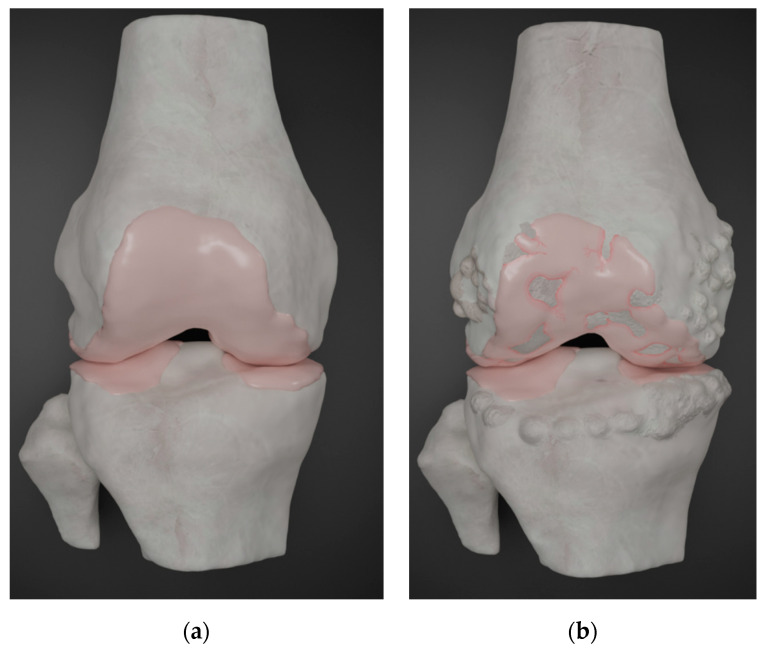
Articular joint structure. (**a**) Bone and cartilage of a healthy tibiofemoral joint (**b**) Simulation of cartilage degeneration and bone spur formation in an osteoarthritic knee.

Normally, synovial joints in the body move effortlessly, gliding across each other [10]. This is due in part to the unique properties of the synovial fluid secreted by the cells of the inner lining of the joint capsule and its ability to reduce the friction that is generated by movement at the joint surfaces [5,10,11]. The articular hyaline cartilage at the joint surfaces maintains the proper structure, function, and stability of the synovial joints [10]. Unfortunately, chondrocytes have limited potential for replication, a factor that contributes to the limited intrinsic healing capacity of cartilage in response to injury. Chondrocyte survival depends on an optimal biochemical and mechanical environment [5,12], Localized stress, wear, and tear lead to degeneration of the cartilage and may also lead to the activation of osteoblasts, resulting in new anomalous bone formation [13,14] (Figure 1). Opportunities for prevention or slowing the progression of degradation may exist if the disease is detected during its early stages. If left untreated, however, OA can lead to complete loss of joint cartilage, severe pain, restricted range of joint and limb movement, and altered reshaping of the bones within the joint [15,16,17].

OA, characterized by joint stiffness, tenderness, swelling, and pain, is not a single disease per se [14]. It is, rather, a complex disease consisting of a group of conditions with multiple pathways that all result ultimately in progressive, irreversible joint failure as the outcome. Many susceptibility factors play a part in the pathogenesis of OA, such as age, gender, and weight [18,19,20]. Old age, female gender, repetitive joint stress, injury, joint laxity, high body weight, and osteoporosis all contribute to OA [21,22]. With the increasing life span of the population in addition to the epidemic of obesity, the number of people affected by joint degeneration is expected to increase substantially [23,24,25]. Severe OA can prevent a person from being able to work, especially if their work involves putting stress on a specific joint for an extended duration. OA can result in significant physical, mental, emotional, and social challenges as well [26,27,28].

Despite nonsurgical and surgical interventions, there is currently neither a cure for this disease nor a means to halt its inevitable progression [22,29]. Autologous chondrocyte transplantation has shown promise in clinical treatment, however, the process involves the harvest, culture, and transplant of cells grown in a monolayer (2D culture) [30,31,32,33]. Under these culture conditions, the risk of dedifferentiation of the chondrocytes and an altered phenotype is a major concern of tissue engineering [34].

## 2. Current Treatment Options and Limitations

The progressive erosion of articular cartilage is a prevalent symptom of OA [35]. Articular cartilage is made up of chondrocytes embedded within a collagenous extracellular matrix (ECM) [36]. Disruption of the articular cartilage prevents pain-free movement and affects the load-bearing abilities of the joint. Current treatments for OA can include intra-articular joint injections of steroids and arthroscopic lavage with debridement; however, these may have short-term benefits without long-term benefit to the cartilage [22,37,38]. Anti-inflammatory and analgesic drugs may help to address some of the symptoms of OA, but they do not tackle the fundamental pathologies involved in the inexorable degenerative process, in addition to having significant side effects associated with prolonged use [39,40,41,42]. Surgery and joint replacement, if possible in the early stages of OA, are expensive and may need to be performed multiple times during an individual’s life [22,43]. Alternative modalities of treatment may well be helpful in the short term, but may not be a sustainable strategy for OA treatment and potential prevention [44,45].

The best way to combat the root cause of joint degeneration in OA may be through the exploration of the various possibilities that genome editing offers [46,47]. There is increasing evidence that genetic and epigenetic modifications play a substantive role in OA; however, it has been difficult to separate the individual effects from the combined effects of genetic and environmental factors acting together to cause progressive joint degeneration [48,49,50,51]. Characterizing and analyzing the genetic factors underpinning the pathology of OA may provide viable options for the diagnosis, prognosis, and development of novel treatment targets for future personalized biological therapies.

## 3. CRISPR/Cas

Several gene therapy systems such as viral, engineered scaffold, and other approaches hold promise [52]. However, while such systems hold promise, the advantages are offset by some potential disadvantages. Here we focus on the CRISPR/Cas9 system, a very powerful gene therapy tool.

CRISPR/Cas is a novel, versatile, and promising genome-editing technique that is opening up new avenues and possibilities in the effective treatment of OA [53] and other degenerative joint diseases [54,55]. CRISPR is an acronym for “clustered regularly interspaced short palindromic repeats”, discovered through investigation of the prokaryotic adaptive immune system. It was identified as an effective system used by bacteria and archaea to remember infecting agents such as phage viruses and destroy them upon subsequent exposure, similarly to the memory cells in the human immune system. When paired with different proteins, specifically enzymes such as Cas, that are produced naturally by the prokaryotic cells, the CRISPR system can be used to make deletions, insertions, substitutions, or other changes at specific sites of the prokaryotic and eukaryotic genome [56,57]. From finding a cure for cancer, to treating sickle-cell disease, to growing drought- and pest-resistant crops, CRISPR has many exciting possibilities and potential in several fields [58].

**Figure 2:**
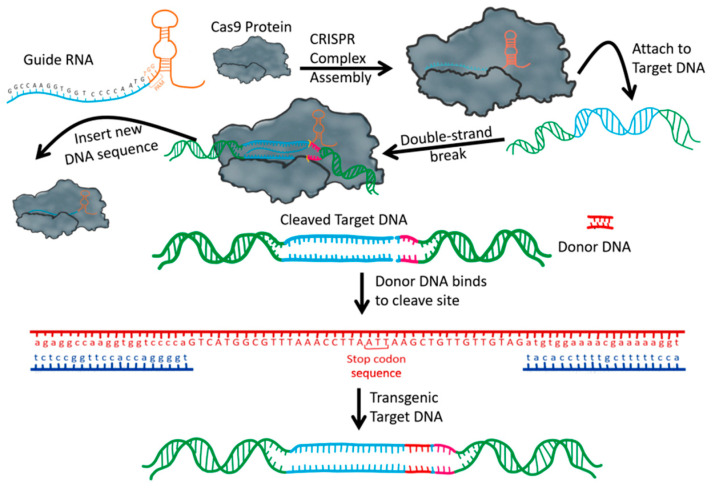
CRISPR/Cas mechanism. Trans-activating RNA (orange) with CRISPR RNA (blue) the guide RNA. The guide RNA assembles with the Cas9 protein to form the CRISPR complex. Using the guide RNA for specificity, the CRISPR complex binds to the target DNA. Transgenic DNA can be inserted using homology arm inserts.

The prokaryotic CRISPR/Cas system has three main components: a Cas nuclease, a crRNA (CRISPR RNA), and a tracrRNA (trans-activating crRNA). In bacterial cells, the CRISPR/Cas system works by recognizing the invading bacteriophage DNA, chopping it up into several pieces, and incorporating them into its DNA. These CRISPR pieces are then transcribed, generating a crRNA and a tracrRNA to create a double-stranded RNA structure that can recruit the Cas proteins. When the offending phage is encountered again, the CRISPR/Cas system is directed to a specific location on the foreign DNA because of a protospacer adjacent motif (PAM) short nucleotide base sequence which is upstream of the crRNA targeted sequence. The Cas protein is programmed to be the “molecular scissors” of the system which carries out the cutting, splicing, and any other editing that is desired [59,60,61]. The therapeutic application of CRISPR/Cas is illustrated in Figure 2.

The most widely used of the six major types of CRISPR/Cas system, the Type II system, is derived from the *Streptococcus pyogenes* bacteria. It uses the Cas9 protein because of its wide working range and efficiency [62]. The CRISPR/Cas9 system that is used currently by researchers and scientists worldwide consists of a single guide RNA (sgRNA) with a binding end (analogous to tracrRNA) for the Cas9 nuclease to attach and a targeting end (similar to crRNA) with nucleotide base pairs that are complementary to the DNA sequence that is meant to be edited (Figure 2). Only the targeting end of the sgRNA needs to be synthesized for any specific targeting sequence, while the binding end does not need to be redesigned every time, thus reducing the time needed to get the tool ready for editing. Cas9 recruitment to the exact DNA target sequence is mediated by the sgRNA. Cas9-induced double-stranded breaks (DSBs) are repaired either by spontaneously nonhomologous end joining (NHEJ) or by homology-directed repair (HDR) using a synthetic donor DNA template [63]. CRISPR/Cas RNA-guided DNA endonuclease genome targeting is much easier to design and apply when compared to other available site-specific editing tools using engineered nucleases such as transcription activator-like effector nuclease (TALENS) and zinc finger nucleases (ZFNs), which are controlled by protein–DNA interactions. CRISPR/Cas is also much more cost- and time-effective because researchers only need to code for a small section of sgRNA [64].

## 4. Mesenchymal Stem Cells and Tissue Regeneration

Tissue regeneration and self-renewal of articular cartilage, in general, is a very limited process [65]. The avascularity of articular cartilage may hinder progenitor cells access to the site of injured cartilage [66]). It may also limit molecular factors that are vital to extracellular matrix repair and homeostasis [67,68,69,70].

Chondrocytes originate from mesenchymal stem cells [71,72]. Bone-marrow-derived MSCs (BM-MSCs) have much promise in aiding articular cartilage repair due to their proximity to the joint, high differentiation capability, and ability to secrete different growth, anti-inflammatory, and immunomodulatory factors [73,74,75,76,77]. They could affect a clinically relevant improvement in joint pain and function. Additional studies are needed, however, to demonstrate the efficacy of cultured versus noncultured BM-MSCs and the best ways to deliver them into the joint. MSCs derived from fetal cells also have therapeutic properties, but ethical concerns have been raised about using them for treatment and therapeutic applications [78,79]. Additional challenges arise due to the potential of fetal MSCs to differentiate into several different types of cells, which might be more difficult to control and direct [76,80,81]. The capabilities of MSCs are usually age-dependent. MSCs have a short lifespan but can secrete paracrine factors that may be beneficial in tissue regeneration [81,82]. In addition to BM-MSCs, MSCs derived from other sites such as adipose tissue can be isolated, expanded, characterized, and used to regenerate cartilage [83,84]. However, MSCs tend to form mechanically inferior fibrocartilage instead of the glassy, hyaline cartilage that covers the ends of bones at articulating joints [85].

## 5. Extracellular Vesicles

**Figure 3:**
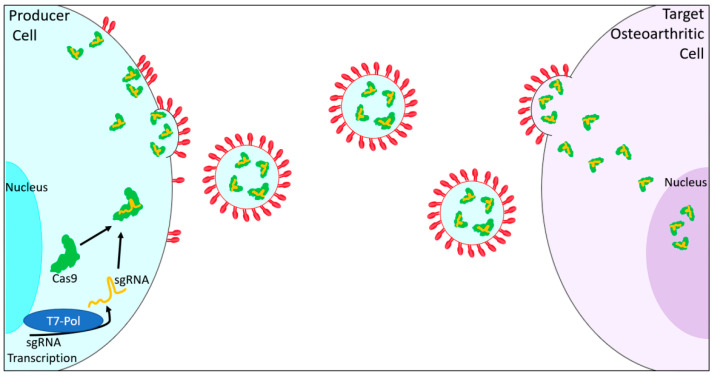
Extracellular vesicle delivery of CRISPR/Cas9 in the treatment of OA. Inside a producer cell (**left**), engineered OA-targeted sgRNA transcription may occur. SgRNA combines with Cas9 to form CRISPR/Cas9 sgRNA complexes. CRISPR/Cas9 complexes load into extracellular vesicles with fluorescent tags containing a dimerization domain compatible with a dimerization domain in engineered CRISPR/Cas9 complexes. These fluorescent tags also contain targets for the target osteoarthritic cell (**right**). Loaded EVs attach to target cells and unload the CRISPR/Cas9 complexes, which are then transported to the nucleus to perform gene modification.

Genome-editing factors, packaged in engineered CRISPR/Cas9 complexes, can be enclosed in extracellular vesicles (EVs), for delivery to specific target cells [86,87]. EVs are composed of cellular constituents such as lipids, proteins, RNA, and DNA [86,88]. EVs may cross the blood–brain barrier, target cells in vivo, and protect their components from degradation in the circulatory system [87,89]. Their function is dependent upon their origin, and EVs derived from MSCs could have the potential to deliver contents to OA cells [90,91,92,93], as shown in Figure 3 [85,94,95]. The use of exosomes as a nonselective cell system may have limitations, including the delivery of contents to unintended cell targets. Possible solutions to this challenge include the design of targeted exosomes, as suggested by Bellavia and colleagues [96].

Exogenous-cell-based therapy is gaining traction for the regeneration of articular cartilage over stimulation therapy such as electric stimulation of endogenous cartilage growth factors [97]. Exogenous cell therapy can be done through the delivery of chondrocytes (either autologous or allogeneic), mesenchymal stem cells (MSCs), or extracellular vesicles [98]. MSCs are used for regenerative medicine due to their ability to promote regeneration based on environmental signals at sites of injury. As inhibitors of the immune system and multilineage differentiators, MSCs are an important alternative cell source for articular cartilage repair and regeneration [97]. MSCs derived from the synovial membranes of joints have been shown to be more effective in terms of articular cartilage formation in in vitro studies when compared to MSCs from other tissues, joint and nonjoint [99,100].

In vitro studies performed in mice showed that microparticles and exosomes, which are EVs derived from MSCs, exerted similar chondroprotective and anti-inflammatory functions, protecting mice from developing osteoarthritis and reproducing the main therapeutic effect of reducing symptoms [101,102]. Thus, a combinatorial approach to the treatment of OA may be feasible.

## 6. Potential CRISPR/Cas9 Molecular Targets for the Treatment of Osteoarthritis

Cell therapy has great potential to help treat OA, but inflammation can prevent new articular cartilage from forming after the introduction of stem cells. Inflammation and inflammatory modulators must be addressed in the treatment of OA, and these inflammatory modulators may serve as targets for CRISPR/Cas9 strategies [103]. Table 1 identifies potential targets for CRISPR/Cas9 editing and the laboratories that are making progress in the use of CRISPR/Cas9 techniques in OA treatment.

IL-1β is a pro-inflammatory cytokine secreted primarily by neutrophils. IL-1β induces the expression of many OA-related genes and other cytokines, including tumor necrosis factor-alpha (TNFα) [104]. Current OA therapies target TNFα, however, deleterious side effects occur due to TNFα’s role in facilitating many other functions [105,106]. Human articular cartilage (hAC) exposed to TNFα displays increased levels of expression of interleukin IL-1β [105,106]. Karlsen and colleagues, in their 2016 study, were able to silence the IL-1β cytokine receptor (IL1-R1) in hACs to determine its effect on inflammation and the redifferentiation potential of the hACs after exposure to the interleukin IL-1β. The hACs were isolated from cartilage, and CRISPR/Cas9 was used to knock out the IL1-R1 receptor and insert a puromycin-resistance gene to allow the selection of the knockout cells. The colonies of knockout cells were expanded and exposed to recombinant IL-1β and TNFα to assess their response. The results showed that the addition of recombinant IL-1β increased inflammation to high levels in the control group, as expected. However, in the knockout group, exposure to recombinant IL-1β did not cause measurable inflammation. Therefore, the therapeutic knockdown of IL1-R1 in articular cartilage cells in vitro prior to re-injection into the body may improve cell-therapy results [106].

Recent gene-editing efforts have targeted cellular senescence. Ren and colleagues found that by targeting CBX4, cellular senescence could be alleviated, with positive outcomes for OA [116]. FOXD1 is a transcription factor that can be regulated by YAP. Recent research indicates that the upregulation of FOXD1 by YAP may hold promise for OA treatment by alleviating senescence [117]. Further studies that focused on connexin 43 modulation were able to demonstrate that the attenuation of cellular senescence could promote the regenerative capacity of cells and improve tissue quality in OA [115].

Degradative enzymes such as matrix metalloproteinases (MMP) play important roles in joint health. Seidl and colleagues utilized CRISPR/Cas9 to modify the MMP13 levels in human chondrocytes and found that by reducing the level of MMP activity, cells were able to accumulate higher levels of the beneficial type II collagen to strengthen the extracellular matrix of the articular cartilage [114].

## 7. Additional Emerging Targets for the Treatment of Osteoarthritis Using CRISPR/Cas9

While a significant focus is placed on cartilage and the chondrocytes that maintain articular cartilage, there is wide agreement that OA is not simply a disease of the cartilage, but rather of the entire joint, and all of the specific tissue types within the joint play essential roles. Osteocalcin, a small protein hormone secreted by the osteoblasts of the bone, has been studied recently for its endocrine functions, which impact several physiological processes. Lambert and colleagues found in their 2016 study that by applying CRISPR/Cas9 technologies to osteocalcin expression, they were able to improve bone biomechanics and increase the trabecular bone in a rat model system [107].

Additional potential targets for therapy have been identified using CRISPR/Cas9 technology. For example, CRISPR/Cas9 knockout of hyaluronan synthase 2 (HAS2) in rat chondrocytes demonstrated the importance of the glycosaminoglycan hyaluronan, for the retention of aggrecan, a proteoglycan necessary for the functional integrity of the articular cartilage [109]. CRISPR/Cas9 can, therefore, provide fundamental information about the molecular mechanisms required for healthy joint tissue in addition to potential use as a direct treatment.

## 8. Limitations and Future Considerations

The CRISPR/Cas9 system is being used in a wide range of applications and many studies. However, notwithstanding its meteoric rise in a short period of time and its potential applications in medicine and beyond, it, like other genome-editing tools, does come with limitations and concerns, ethical and otherwise. One of these limitations is the effective targeting range, as the sgRNA can only bind to a region near a specific PAM sequence on the DNA. The PAM sequence for Cas9 is 5′-NGG-3′, where “N” can be any nucleotide base, but the third base must be G. This can greatly reduce the potential target locations available to make DNA edits such as insertions or deletions. In experiments conducted by Nishimasu and colleagues, a Cas9 with a more relaxed preference for the PAM third base resulted in the recognition of an NGD PAM instead of an NGG PAM requirement. This effectively increased the potential targets for Cas9 nuclease, as the NGD sequence occurs more frequently in human DNA than NGG sequences. The engineered Cas9 in this instance had a wider range and increased cleavage specificity, reducing instances of off-target incisions. The new Cas, termed SpCas9-NG, can bind to A, G, or T in the 3rd base of the PAM sequence [118].

MicroRNAs (miRNAs), small, non-coding RNA molecules, may help to regulate inflammation, promote MSC differentiation, and ensure the homeostasis of cartilage [119]. As a key factor in epigenetic regulation, miRNAs can change the gene expression without modifying the sequence of the DNA that encodes proteins [120]. This may be a much safer way to modulate gene expression since the genome sequence does not change, and the gene expression pattern may still be inheritable from one cell generation to the next [85]. miRNAs may be used in combination with CRISPR/Cas9 and EVs to design patient-specific approaches to the treatment of OA [103].

## 9. Conclusions

Investigators and practitioners the world over are working toward a better understanding of the basis of degenerative joint diseases such as OA. Devising ways to alter or modify the relevant genes impacting the joint articular cartilage may lead to the development of successful, safe, and effective therapies to halt the progress, treat, or even prevent the occurrence of OA and other debilitating joint disorders in humans. CRISPR/Cas9, MSCs, EVs, and miRNAs may all play key roles in future treatments.

## Figures and Tables

**Table 1:** Potential CRISPR/Cas9 molecular targets for CRISPR/Cas treatment of osteoarthritis.

Gene Symbol	Gene Name	Function	Reference
IL1-β	*Interleukin 1 beta*	Inflammation	Karlsen, 2016 [106] Zhao, 2019 [53] Guilak [73]
IL1-R1	*Interleukin 1 beta receptor*	Inflammation	Karlsen, 2016 [106]
BGLAP	*Osteocalcin*	Trabecular bone formation	Lambert, 2016 [107]
miR-140	*Micro RNA 140*	Chondrocyte homeostasis	Asahara, 2016 [108]
Has2	*Hyaluronan synthase 2*	Chondrocyte accumulation of aggrecan	Huang, 2016 [109]
sTNFR1α	*Soluble Tumor necrosis factor receptor 1*	TNF antagonist	Brunger, 2017 [110]
IL1RA	*Interleukin 1 bets receptor antagonist*	IL-1 beta antagonist	Brunger, 2017 [110]
PRG4	*Lubricin*	Joint lubrication	Khakshooy, 2017 [111]
Runx2	*Runt Related Transcription Factor 2*	Osteoblast differentiation	Rice, 2018 [112]
Hrdl	*E3 ubiquitin-protein ligase hrd-like protein 1*	Protein turnover and proteasomal degradation	Ye, 2018 [113]
Mmp13	*Matrix metalloprotein 13*	Tissue degradation	Seidl, 2019 [114] Zhao, 2019 [53]
Cx43	*Connexin 43*	Gap junction protein	Varela-Eirín M, 2018 [115]
NGF	*Nerve growth factor*	Pain sensitivity	Zhao, 2019 [53]
Cbx4	*Chromobox 4*	Nucleolar homeostasis	Ren, 2019 [116]
Foxd1	*Forkhead box D1*	Transcription factor	Fu, 2019 [117]
YAP	*Yes-associated protein 1*	Mechanosensing transcription factor	Fu, 2019 [117]

## Abbreviations

OA	Osteoarthritis
CRISPR	Clustered Regularly Interspaced Short Palindromic Repeats
Cas	Cas nuclease
crRNA	CRISPR RNA
tracrRNA	trans-activating crRNA
PAM	protospacer adjacent motif
sgRNA	single guide RNA
MSC	Mesenchymal stem cells
miRNA	Micro RNA
EV	Extracellular vesicles
IL-1β	Interleukin 1 beta
TNFα	Tumor Necrosis Factor
Hac	Human articular cartilage
IL1-R1	IL-1β cytokine receptor 1
BGLAP	Osteocalcin
Has2	Hyaluronan synthase 2
PRG4	Lubricin
Runx2	Runt Related Transcription Factor 2
Hrdl	E3 ubiquitin-protein ligase hrd-like protein 1
Mmp13	Matrix metalloprotein 13
Cx43	Connexin 43
Cbx4	Chromobox 4
Foxd1	Forkhead box D1
YAP	Yes-associated protein 1
NHEJ	Non-homologous end joining
HDR	Homology Directed Repair
TALENs	Transcription activator-like effector nucleases
ZFN	Zinc finger nucleases
